# Factors associated with diarrheal disease among children aged 1–5 years in a cholera epidemic in rural Haiti

**DOI:** 10.1371/journal.pntd.0009726

**Published:** 2021-10-22

**Authors:** Hilary A. Dolstad, Molly F. Franke, Kenia Vissieres, Jean-Gregory Jerome, Ralph Ternier, Louise C. Ivers

**Affiliations:** 1 Harvard Medical School, Boston, Massachusetts, United States of America; 2 Department of Global Health and Social Medicine, Harvard Medical School, Boston, Massachusetts, United States of America; 3 Partners In Health / Zanmi Lasante, Cange, Haiti; 4 Center for Global Health, Massachusetts General Hospital, Boston, Massachusetts, United States of America; Emory University, UNITED STATES

## Abstract

Diarrheal illness is a major cause of morbidity and mortality among children in Haiti, and the impact of diarrheal illness was compounded by a cholera outbreak between 2010 and 2019. Our understanding of risk factors for diarrhea among children during this outbreak is limited. We conducted a secondary analysis of data collected as part of a cholera vaccine effectiveness study to identify factors associated with medically attended diarrhea among children in central Haiti from October of 2012 through November of 2016. We identified 47 children aged one to five years old who presented to medical clinics with acute, watery diarrhea, and 166 matched controls who did not have diarrhea, and we performed conditional logistic regression to identify factors associated with diarrhea. Discontinuing exclusive breastfeeding within one month of birth was associated with increased risk of diarrhea (RR 6.9, 95% CI 1.46–32.64), and diarrhea was inversely associated with reported history of supplementation with vitamin A (RR 0.05, 95% CI 0.004–0.56) and zinc (reported among 0% of cases vs. 17% of controls). Because of the concordance in supplementation patterns, it was not possible to attribute the association to vitamin A or zinc independently. While having a respondent who correctly identified ≥3 means of avoiding cholera was associated with reduced risk of diarrhea (RR 0.43, 95% CI 0.19–1.01), reported household sanitation practices and knowledge of cholera were not consistently associated with risk of diarrhea. These findings support ongoing efforts to reduce barriers to breastfeeding and promote pediatric supplementation with vitamin A and zinc in Haiti. Given the reduced efficacy of current oral cholera vaccines (OCV) among children, the results reinforce the importance of breastfeeding and micronutrient supplementation in preventing all-cause pediatric diarrheal illness generally and during cholera outbreaks.

## Author summary

Diarrheal diseases are leading causes of illness and death among children throughout the world, and children in Haiti were particularly impacted by diarrhea during the cholera outbreak that started in 2010. Between 2012 and 2016, data were collected as part of a case-control study of oral cholera vaccine (OCV) effectiveness in Haiti. We analyzed data from that study to identify factors associated with diarrheal illness, including cholera and non-cholera diarrhea, among children ages one through five years old. We found a direct association between longer duration of exclusive breastfeeding and supplementation with vitamin A and zinc and a reduced risk of diarrhea. These findings shed light on potentially important components of efforts to reduce pediatric diarrheal illness in Haiti generally, and to reduce pediatric diarrhea in the context of cholera outbreaks in Haiti and elsewhere.

## Introduction

Diarrheal illness is a leading cause of human morbidity and mortality globally [1]. Among children, diarrheal illness has a particularly profound impact, accounting for one in ten deaths in children younger than five years old [2]. In Haiti, where the under-five mortality rate is 75 per 1000 live births, 34% of pediatric hospitalizations at four large hospitals were attributed to diarrheal illness between 2010 and 2012 [3, 4]. The impact of diarrheal illness in Haiti specifically was amplified given the introduction of cholera to Haiti in 2010. The lack of local immunity, limited access to clean water, and infrastructural damage after a massive earthquake in January of 2010 contributed to the rapid development of a severe epidemic, which accounted for almost half of all cholera deaths reported to the World Health Organization (WHO) globally in 2010 and 2011 [5–8]. Following its introduction, cholera became an important cause of pediatric diarrhea in Haiti, amplifying the already high morbidity caused by other etiologies of diarrhea such as rotavirus [4, 9]. Cholera infections in children under five years of age accounted for more than 30,000 hospitalizations between 2010 and 2012 in Haiti [5].

Among other efforts to control diarrheal illness, vaccines for both rotavirus and cholera have been introduced to Haiti. In 2009, the WHO recommended rotavirus vaccination for all children under age 32 weeks, and Haiti added rotavirus vaccine to its national immunization program in 2014 [10, 11]. Although its effectiveness is less pronounced in countries with high child mortality rates, studies in Latin America and the Caribbean have shown that the vaccine is effective in preventing diarrhea-related hospitalizations [12, 13]. Oral cholera vaccines (OCVs) have similarly shown effectiveness in reducing the risk of cholera, and these vaccines were introduced to Haiti in 2012 as part of efforts to control the outbreak [14, 15]. However, OCVs have demonstrated reduced efficacy among the pediatric population [16, 17].

Given the suboptimal effectiveness of OCVs in children and the large proportion of diarrheal disease that cannot be prevented by vaccination, identifying and intervening on other risk factors is critical to reducing morbidity and mortality from diarrheal disease in children. While a number of studies have investigated risk factors for diarrheal illness in children in other countries, research specific to risk factors for the pediatric population in Haiti is limited [18–21]. Understanding specific risk factors for diarrheal illness by context is an essential element of prevention.

Previous studies globally have found that children’s nutritional and breastfeeding history, along with sanitation and hygiene factors, can influence pediatric risk of diarrhea. Exclusive breastfeeding, particularly among infants 0–5 months of age, has been shown to decrease diarrheal incidence and mortality [22]. Malnutrition has been associated with increased duration and severity of diarrhea, as have specific micronutrient deficiencies, including vitamin A and zinc [23–26]. Previous studies have also demonstrated the association between water, sanitation, and hygiene (WASH) factors and diarrheal risk, including evidence showing that point-of-use water treatment reduces diarrheal risk in adults and children, although these studies can be subject to bias given the inherent difficulty in blinding subjects of interventions [27–31]. Diarrhea is a major contributor to child malnutrition, and some research suggests that WASH interventions reduce diarrhea as well as child malnutrition as measured by benefits on growth, although recent large-scale randomized controlled trials of WASH have found limited evidence of effect [32–34].

We aimed to identify factors associated with all causes of medically attended diarrhea (i.e., cholera and non-cholera diarrhea) in children aged one to five years in Haiti. We analyzed data that were collected from 2012 through 2016 as part of an OCV effectiveness study. Based on the risk factors identified in the general population in Haiti, and in children in other parts of the world, we hypothesized that possible associated factors would include breastfeeding and nutritional history, hygiene and sanitation practices and knowledge (both general and specific to cholera), and household economic status [35–39].

## Methods

### Ethics

The Partners Institutional Review Board provided ethical approval for this analysis (Partners protocol #2012P000393, Boston, MA, USA). The Haitian National Bioethics Committee (Port-au-Prince, Haiti) also provided ethical approval for the initial studies. Written informed consent was obtained from a parent or guardian.

### Study context

We performed a secondary analysis of two case-control studies conducted between October of 2012 and November of 2016. The first of these studies evaluated the effectiveness of two oral cholera vaccine campaigns in rural Haiti, and the second study assessed the likelihood of bias in the vaccine effectiveness study [14, 17, 40, 41]. The vaccine effectiveness study examined the relationship between receipt of OCV and medically attended cholera, whereas the bias indicator study analyzed the relationship between cholera vaccination and non-cholera diarrhea. In the vaccine effectiveness case-control study, cases were individuals with medically attended cholera presenting to a participating study site, and in the bias indicator study, cases were individuals with non-cholera medically attended diarrhea who presented to the same study sites. The two primary studies were conducted simultaneously and employed the same survey tools and interview protocols. With regard to the findings of the present analysis, ’diarrhea’ refers to medically attended, acute, watery diarrhea.

### Study eligibility

Study participants were residents of the vaccine catchment areas in the Artibonite or Central Departments who were eligible to receive OCV (whether they had received vaccine or not). Vaccination eligibility criteria included age of at least 12 months and residence in the region at the time of the vaccination campaign.

For this analysis, cases were children aged one to five years who presented to one of three study site clinics with acute, watery diarrhea, defined as three or more loose, non-bloody, liquid stools in a 24-hour period with an onset of three days or fewer before presentation. Participants’ stool samples were tested by the Crystal VC rapid test and by culture. A stool sample positive for *V cholerae* 01 by culture defined a participant’s status as a case due to cholera, whereas a stool sample negative for cholera by culture defined a participant’s status as a case due to non-cholera diarrhea. We combined cholera and non-cholera diarrhea cases to study all causes of diarrhea.

Controls were children aged one to five years who had not sought treatment for diarrhea between the first day of study enrollment and the day when their corresponding case experienced symptom onset. Controls were matched to cases by location of residence, age range between 1–4 years or 5–15 years, and enrollment time within two weeks of the case. For the present analysis, only controls aged 1–5 years were included. When possible, controls of the same sex as the case were selected. When more than one eligible control was available in a household, an individual of the same sex as the case was selected when possible. If more than one eligible control was available but all were a different sex than the case, the one most closely matching the case in age was selected. To identify controls, study workers approached the homes nearest to the case’s home until up to four controls were identified. If the patient lived within a cluster of multigenerational families, also known as a “lakou,” we sought controls outside the “lakou” in which the case resided.

### Interview procedures

Native Haitian Creole-speaking study staff used standardized forms to interview a guardian or family member proxy for each child participant in the original studies. Interviewers asked questions regarding participant demographics, household characteristics, dietary risk factors, hygiene, respondent knowledge of cholera, and the participant’s oral cholera vaccination status. Interviewers asked questions regarding breastfeeding for the subset of children aged three years old and under. The questions related to knowledge about cholera included, “How can a person get cholera?” “How can a person avoid cholera?” “What are the ways to treat water that you drink?” and “When should a person wash their hands?” Interviewers asked respondents to provide as many answers to these questions as possible, and we assessed the association between the respondent’s ability to provide three or more correct answers to each question and diarrhea in the pediatric participant who lived in the household [42].

### Statistical analysis

Our primary outcome of interest was diarrhea. We used conditional logistic regression adjusted for matching factors to calculate odds ratios, 95% confidence intervals, and *P* values for variables of interest. Because the outcome of medically attended diarrhea was rare in the population, the odds ratio approximates the risk ratio, and we refer to results of logistic regression as relative risks [43].

Given our specific interest in breastfeeding and nutritional factors, we first created seven multivariable models to assess the association of each of the following independent variables with diarrhea: vitamin A supplementation within the past six months, zinc supplementation within the past six months, duration of any breastfeeding in months, any breastfeeding for six months, duration of exclusive breastfeeding in months, exclusive breastfeeding for six months, and exclusive breastfeeding for less than one month. Because data for these variables were available for different subsets of the population (e.g., we asked about breastfeeding only for participants aged three and under), each of these factors of interest required its own multivariable model. We adjusted for expected confounders between these factors and diarrhea, identified a priori: participant age, relationship with respondent, reported cholera vaccination, and earthen floor in the home (i.e., an indicator of poverty) [44]. Data were missing if the respondent did not know the child’s supplementation or breastfeeding history. Primary analyses were complete case. In order to assess the impact of missing data on the results, we conducted two sensitivity analyses, one using the missing indicator method, and another in which we interpreted missing values as negative for the exposures of interest. In the missing indicator approach, a variable was included in the model which indicated whether data were missing for a given participant [45].

We created an additional model to examine general household and interview respondent characteristics associated with risk of diarrhea in children. In order to identify factors to include in this analysis, we conducted an exploratory review of physiologically or socially plausible factors identified in a literature review. Factors identified in this univariable analysis at a significance level of *P* ≤ 0.10 were then included as independent variables in a multivariable model, with diarrhea as the dependent variable. A cut-off of *P* ≤ 0.10 was selected because it would identify the strongest risk factors while allowing us to maintain a parsimonious model. We used this approach due to the exploratory nature of the aim, the need for parsimony given a small number of observations, and careful consideration to plausibility and causal structure. We included age in all multivariable models to adjust for residual confounding, since cases and controls were matched in broad age groups. We used SAS Studio to perform all analyses (SAS Institute, Cary, NC).

## Results

### Characteristics of study participants

Between October of 2012 and November of 2016, there were 47 cases of diarrhea in children, with a total of 166 matched controls, resulting in a total sample size of 213 (Table 1). Of the 47 cases, 25 cases were due to cholera, and 22 cases had non-cholera diarrhea. The median age was 3 years for cholera cases, 3.5 years for non-cholera diarrheal cases, and 3 years for controls.

**Table 1:** Respondent and participant characteristics.

	Cholera Cases (N = 25) n (%)		Non-cholera Diarrhea Cases (N = 22) n (%)		Controls (N = 166) n (%)	
**Household and Participant**						
Female sex	13	(52%)	10	(45%)	82	(49%)
Age in years (Median and IQR)	3	(3–4)	3.5	(2–4)	3	(3–4)
Reported receipt of cholera vaccine	18	(72%)	14	(64%)	125	(75%)
Household size, Median and IQR	6	(5–7)	6	(5–7)	6	(4–7)
Respondent is mother	19	(76%)	16	(73%)	116	(70%)
Household has electricity^*a*^	6	(24%)	7	(33%)	44	(27%)
Number of children age <5 years old in household (Median and IQR)^*b*^	1	(1–2)	1	(1–2)	1	(1–2)
**Household floor type**						
Earth	19	(76%)	17	(77%)	116	(70%)
Cement	6	(24%)	5	(23%)	49	(30%)
Wood	0	(0%)	0	(0%)	1	(1%)
**Respondent occupation**						
Agriculture	14	(56%)	11	(50%)	85	(51%)
Commerce	17	(68%)	11	(50%)	65	(39%)
Artisan, fishing, small job, or other	1	(4%)	2	(9%)	9	(5%)
Not working	2	(8%)	2	(9%)	25	(15%)
**Respondent knowledge**						
≥ 3 correct modes of cholera transmission	9	(36%)	4	(18%)	57	(34%)
≥3 correct means of avoiding cholera	10	(40%)	5	(23%)	75	(45%)
≥3 correct means of treating water	11	(44%)	12	(55%)	63	(38%)
≥3 correct answers on when to wash hands	10	(40%)	14	(64%)	89	(54%)
Household buys water	2	(8%)	4	(18%)	20	(12%)
Always treat water	13	(52%)	12	(55%)	74	(45%)
30 minutes or more on foot to river	10	(40%)	6	(27%)	45	(27%)
Household ran out of firewood in past week	9	(36%)	6	(27%)	46	(28%)
**Toilet type**						
Latrine	18	(72%)	15	(68%)	99	(60%)
Open garden	7	(28%)	7	(32%)	66	(40%)
Flush toilet	0	(0%)	0	(0%)	1	(1%)
**Number that share toilet** among those who use latrine (Median and IQR)	10	(9–20)	7	(6–10)	10	(7–10)
**≥ 1 unimproved domestic water source** ‖	16	(64%)	13	(59%)	104	(63%)
**Nutritional Factors**						
Received vitamin A within last 6 months^*c*^	1	(6%)	3	(30%)	28	(28%)
Received zinc within last 6 months^*d*^	0	(0%)	0	(0%)	15	(17%)
**Duration of any breastfeeding** in months (Median and IQR)^*e*^	18	(12–21.5)	18	(16–24)	18	(16–22)
Any breastfeeding for ≥ 6 months	14	(88%)	9	(90%)	83	(93%)
**Duration of exclusive breastfeeding** in months (Median and IQR)^*e*^	2.5	(1–3)	3	(1–6)	3	(1–6)
Exclusive breastfeeding for 6 months	3	(19%)	4	(40%)	28	(31%)
Exclusive breastfeeding for ≤ 1 month	7	(44%)	4	(40%)	26	(29%)

‖ River, source water, bottled water, well, truck

*a* n = 25, 21, 166

*b* n = 24, 21, 160

*c* n = 18, 10, 100

*d* n = 12, 7, 90

*e* n = 16, 10, 89

Sixty percent of cases and 60% of controls (n = 128) were able to provide responses regarding whether the child had received supplementation with vitamin A within the last six months. Among these, the rate of vitamin A receipt was 6% (one of 18) for cholera cases, 30% (three of 10) for non-cholera cases, and 28% (28 of 100) for controls. Respondents for 40% of cases and 54% of controls (n = 109) were able to provide information regarding whether the child had received supplementation with zinc within the past six months; none of the 19 cases and 17% (15 of 90) of controls reported receipt of zinc. Of the 32 total participants who reported receipt of vitamin A, 15 also reported receipt of zinc. All 15 of the participants who reported receipt of zinc also reported receipt of vitamin A, while five participants who reported that they did not receive zinc did report receipt of vitamin A.

Among the children aged three and under, respondents were able to provide responses about breastfeeding for 16 out of 17 (94%) cholera cases, all 10 non-cholera diarrhea cases, and 89 out of 91 (98%) controls. The rate of exclusive breastfeeding for six months was 19% (three of 16) among cholera cases, 40% (4 of 10) among non-cholera cases, and 31% (28 of 89) among controls. The frequency of discontinuing exclusive breastfeeding within one month after birth was 44% (seven of 16) among cholera cases, 40% (four of 10) among non-cholera cases, and 29% (26 of 89) among controls. None of the children were currently breastfed. Table 1 includes additional descriptive characteristics of cases and controls.

### Nutritional factors associated with childhood diarrhea

#### Univariable analysis

Among children whose respondents provided responses regarding receipt of vitamin A and zinc, cases were less likely than controls to have recent nutritional supplementation (Table 2). Fourteen percent of cases reported receipt of vitamin A within the past six months, compared to 28% of controls (RR 0.20, 95% CI 0.04–1.04, *P* = .06). None of the cases reported receiving zinc within the past six months, compared to 17% of controls. Because none of the cases reported zinc supplementation, we were unable to conduct regression analyses for exposure to zinc supplementation.

**Table 2:** Univariable and multivariable analysis of nutritional risk factors for diarrheal illness.

	Cases n (%) or median (IQR)		Controls n (%) or median (IQR)		Unadjusted RR (95% CI) and P Value		Adjusted RR (95% CI) and P Value^¶^	
Received Vitamin A within last 6 months^*a*^	4	(14%)	28	(28%)	0.20 (0.04–1.04)	0.06	0.05 (0.004–0.56)	0.02
Received Zinc within last 6 months^*b*^	0	(0%)	15	(17%)	†	†	†	†
Duration of any breastfeeding^*c*^ In months (Median and IQR)	18	(13–24)	18	(16–22)	0.99 (.92–1.05)	0.67	0.99 (0.92–1.06)	0.73
Any breastfeeding for ≥ 6 months	23	(88%)	83	(93%)	0.58 (0.12–2.67)	0.48	0.51 (0.10–2.66)	0.42
Duration of exclusive breastfeeding In months (Median and IQR)^*c*^	3	(1–6)	3	(1–6)	0.78 (0.58–1.06)	0.11	0.78 (0.56–1.08)	0.14
Exclusive breastfeeding for 6 months	7	(27%)	28	(31%)	0.52 (0.15–1.85)	0.31	0.59 (0.15–2.26)	0.44
Exclusive breastfeeding for ≤ 1 month	11	(42%)	26	(29%)	3.49 (1.0–12.2)	0.05	6.9 (1.46–32.64)	0.01

¶ Adjusted for age (years), respondent relationship with participant, home has earthen floor, self-reported vaccination status

† Model did not converge because no cases reported receipt of zinc within past six months

*a* Adjusted complete case analysis includes 28 cases and 100 controls

*b* Adjusted complete case analysis includes 19 cases and 90 controls

*c* Adjusted complete case analysis includes 26 cases and 89 controls

Among the children aged three years and under, breastfeeding rates were also lower among cases than controls. While the rate of exclusive breastfeeding for six months was similar among cases and controls (27% versus 31%), cases were more likely to discontinue exclusive breastfeeding after one month or less, with 42% of cases and 29% of controls exclusively breastfed for one month or less (RR 3.49, 95% CI 1.0–12.2, *P* = 0.05).

#### Multivariable analysis

We adjusted the breastfeeding and nutrition factors for participant age, relationship with respondent, reported cholera vaccination status, and earthen floor in household (Table 2). Report of receiving vitamin A within the last six months was inversely associated with diarrhea (RR 0.05, 95% CI 0.004–0.56, *P* = 0.02), and discontinuing exclusive breastfeeding within one month of birth was directly associated with diarrhea (RR 6.9, 95% CI 1.46–32.64, *P* = 0.01).

#### Sensitivity analyses

Results from sensitivity analyses in which we (1) employed the missing indicator method or (2) assumed that children for whom we lacked nutritional exposure data were unexposed showed decreased precision but did not change the overall associations observed in the primary complete case analyses (S1 and S2 Tables).

### Household and respondent characteristics associated with childhood diarrhea

#### Univariable analysis

A lower frequency of respondents for cases (32%) than controls (45%) provided three or more correct answers regarding means of avoiding cholera (RR 0.52, 95% CI 0.24–1.13, *P* = 0.10), whereas a higher proportion of cases (49%) than controls (38%) provided three or more correct answers regarding ways of treating water (RR 2.04, 95% CI 0.95–4.4, *P* = 0.07; Table 3). The percent of cases and controls who provided three or more correct answers regarding modes of cholera transmission and handwashing were similar.

**Table 3:** Univariable and multivariable analysis of general household and participant risk factors for diarrheal illness.

	Cases (N = 47) n (%) or median (IQR)		Controls (N = 166) n (%) or median (IQR)		Unadjusted RR (95% CI) and P Value		Adjusted RR (95% CI) and P Value §	
**Sociodemographic**								
Female sex	23	(49%)	82	(49%)	0.98 (0.51–1.9)	0.96		
House has earthen floor	36	(77%)	116	(70%)	1.61 (0.67–3.9)	0.29		
Household size, Median and IQR	6	(5–7)	6	(4–7)	1.04 (0.87–1.25)	0.64		
Respondent occupation:								
Agriculture	25	(53%)	85	(51%)	1.12 (0.54–2.35)	0.76		
Commerce	28	(60%)	65	(39%)	2.91 (1.39–6.1)	<0.01	3.16 (1.40–7.11)	0.01
Artisan, fishing, small job, or other	3	(6%)	9	(5%)	1.16 (0.26–5.23)	0.85		
Not working	4	(9%)	25	(15%)	0.41 (0.12–1.43)	0.16		
**Knowledge**								
≥ 3 correct modes of cholera transmission	13	(28%)	57	(34%)	0.74 (0.34–1.6)	0.45		
≥3 correct means of avoiding cholera	15	(32%)	75	(45%)	0.52 (0.24–1.13)	0.1	0.43 (0.19–1.01)	0.05
≥3 correct means of treating water	23	(49%)	63	(38%)	2.04 (0.95–4.4)	0.07	2.0 (0.89–4.47)	0.09
≥3 correct answers on when to wash hands	24	(51%)	89	(54%)	0.84 (0.42–1.7)	0.63		
**Food and Water**								
Household buys water	6	(13%)	20	(12%)	1.38 (0.31–6.2)	0.68		
Always treat water	25	(53%)	74	(45%)	1.81 (0.82–3.98)	0.14		
30 minutes or more on foot to river	16	(34%)	45	(27%)	1.83 (0.56–6.02)	0.32		
Household ran out of firewood in past week	15	(32%)	46	(28%)	1.50 (0.53–4.29)	0.45		
**Sanitation and Hygiene**								
Household toilet is latrine	33	(70%)	99	(60%)	2.07 (0.88–4.88)	0.1	1.97 (0.78–4.98)	0.15
Number that share toilet among those who use latrine (Median and IQR)	10	(7–12)	10	(7–10)	1.04 (0.96–1.11)	0.36		
≥1 unimproved domestic water source ‖	29	(62%)	104	(63%)	0.87 (0.30–2.51)	0.79		

§ Adjusted for age (years), respondent makes a living in commerce, ≥3 correct answers on means of avoiding cholera, ≥3 correct answers on means of treating water, and toilet is a latrine. Adjusted analysis includes all 47 cases and 166 controls.

‖ River, source water, bottled water, well, truck

Respondent self-report of making a living by working in commerce was more common among cases (60%) than controls (39%) (RR 2.91, 95% CI 1.39–6.1, *P* < .01). Having a latrine as the main household toilet was also elevated among cases (70% vs. 60%; RR 2.07, 95% CI 0.88–4.88, *P* = 0.10).

#### Multivariable analysis

The final multivariable model for general risk factors included participant age, respondent (i.e., parent or household member) self-report of making a living in commerce, three or more correct answers on means of avoiding cholera, three or more correct answers on means of treating water, and having a latrine as the household toilet (Table 3). Providing three or more correct responses regarding means of avoiding cholera was inversely associated with diarrhea (RR 0.43, 95% CI 0.19–1.01, *P* = 0.05). Respondent self-report of making a living by working in commerce was associated with increased risk of diarrhea (RR 3.16, 95% CI 1.40–7.11, *P* = 0.01).

## Discussion

Our findings highlight the beneficial associations between breastfeeding, vitamin A, and zinc and reduced risk of diarrhea among children aged one to five years in Haiti during a cholera outbreak. We also found an association between increased respondent knowledge of ways of avoiding cholera and reduced risk of pediatric diarrhea, although there was not a consistent pattern between childhood diarrhea and household knowledge or practices. While the data are limited by a small sample size, these findings suggest potential directions for future interventions and research.

We found that rates of exclusive breastfeeding for six months were relatively low among all participants, and diarrheal cases were more likely than controls to have been exclusively breastfed for one month or less. The protective effects of exclusive breastfeeding on childhood diarrheal illness, including cholera, have been shown previously in multiple contexts [22, 46–49]. Passive immunity and reduced exposure to pathogens likely both contribute to this [50]. The WHO recommends exclusive breastfeeding until age six months, but in 2016–2017, only 40% of children under six months old in Haiti were exclusively breastfed, below the 50% target put forth by the WHO [51, 52]. Research in Haiti and elsewhere has shown a complex interplay between social and economic factors and exclusive breastfeeding. Poverty can lead to reduced breastfeeding due to insufficient maternal food, time constraints, and physical and emotional stress [53, 54]. Because poverty has also been shown to be a risk factor for childhood diarrhea, there could be an element of reverse causation in our findings, as factors that make mothers less likely to breastfeed could also predispose their children to diarrheal illness [55]. In addition to poverty, maternal employment has also been associated with reduced exclusive breastfeeding [56, 57]. In Haiti, studies have shown widespread belief in the importance of breastfeeding to promote infant health and development [53, 58]. However, a woman’s decision and ability to breastfeed is multifactorial, and our findings support the need for continued efforts to address the manifold barriers to exclusive breastfeeding in Haiti.

In addition to breastfeeding, our data showed that supplementation with vitamin A and zinc within the past six months was associated with reduced risk of diarrheal illness among children, although the concordance in supplementation patterns limits our ability to attribute the relationship to either zinc or vitamin A independently. Zinc has previously been shown to reduce diarrhea duration, severity and diarrhea-related mortality, likely due to its role in the innate and adaptive immune systems [25, 59, 60]. The protective effects of zinc have been shown both as a diarrheal treatment and as a supplement in settings with high rates of deficiency [61]. Vitamin A has also been shown to reduce diarrhea severity and mortality among children under five years of age [25]. The WHO therefore recommends zinc as an integral component of diarrhea treatment in children, and universal vitamin A supplementation for children ages 6–59 months in countries with high rates of deficiency [62, 63]. Yet as of 2017, the rate of vitamin A supplementation among young children in Haiti was only 17% [64]. In the context of the high burden of diarrheal illness among children in Haiti, our data support efforts to promote pediatric supplementation with vitamin A and zinc.

Additionally, our results suggest the importance of nutrition and breastfeeding as part of cholera outbreak response, both in Haiti and elsewhere. Household food insecurity has been shown by our group to be independently associated with risk of cholera, and food insecurity is also a risk factor for malnutrition and micronutrient deficiencies among children [65, 66]. The associations between food insecurity, malnutrition, and childhood cholera and diarrheal disease emphasize the importance of integrated public health strategies in diarrheal disease control.

We did not find a consistent pattern in the relationship between diarrhea and respondents’ knowledge related to sanitation, hygiene or cholera. While increased respondent knowledge about how to avoid cholera was associated with a reduced risk of pediatric diarrhea, we found no association between respondent knowledge of cholera transmission or hand-washing and pediatric diarrheal risk. Overall, our data show that on three of four questions, respondents for children with diarrhea demonstrated similar or even greater levels of knowledge than respondents for children without diarrhea. Our group’s previous work demonstrated that other factors, such as financial constraints and lack of access to sanitation products, limited respondents’ ability to put their knowledge into practice [42, 67, 68].

Interestingly, some of the hypothesized risk factors, such as reported water treatment practices and water sources, did not appear to be associated with diarrhea in our analysis. This could be explained in part by interactions between the variables, which we were unable to examine because of the sample size, or by lack of heterogeneity in the conditions. Additionally, previous research in Haiti has shown contamination of many improved water sources, and that these sources are often off-site, requiring transport and safe storage [69]. Cost and access have also been demonstrated as significant barriers to effective water treatment [42]. In this context, our findings highlight the challenges to consistently accessing clean water in our study sites in Haiti, and the complexities of measuring access to safe water.

We also found that having an interview respondent who worked in commerce was associated with a 3-fold higher risk of diarrhea in children compared to other livelihoods. One possible explanation for this result is that working in commerce could increase family members’ interpersonal interactions with others, and thus increase risk of household exposure. However, this finding warrants further exploration.

We previously reported that having a latrine as the main household toilet was a risk factor for cholera among individuals in our primary dataset who had received cholera vaccine, and here we observed a high frequency of household latrine use among children with diarrhea, though confidence intervals were wide [35]. This could be explained in part by the number of individuals who shared latrines, which ranged from two to 50 in our study. Higher numbers of people sharing latrines could increase risk of disease transmission via the latrine, especially if hygiene materials and practices were insufficient for the volume of use [70]. Other studies have reported latrine sharing as a diarrheal risk factor in both adults and children [71–73]. Latrine use reduces surface water contamination and is thus important for public health, but ineffective hygiene interventions may introduce individual risk at the latrine [74]. Therefore, these findings support the need to evaluate effectiveness when implementing interventions focused on latrine use.

### Limitations

Among the limitations of this analysis was a relatively small sample size, which limited our ability to identify smaller differences between the case and control groups. The small number of observations also necessitated a parsimonious model, and therefore there may be uncontrolled confounding. While we sought to control for household wealth, the limited amount of socioeconomic data in our dataset also leaves the possibility for residual confounding. We also assessed multiple factors, raising the possibility for chance findings. Because all of the participants who received zinc also received vitamin A, it is not possible to ascribe the beneficial association between diarrheal disease and zinc to zinc alone. Similarly, given overlap in receipt of zinc and vitamin A, concurrent zinc supplementation could confound the findings regarding benefits of vitamin A. Furthermore, because zinc is often given as a treatment for diarrhea, it is possible that children who received zinc supplementation were protected from diarrhea in part due to immunity from prior diarrheal illness. Additionally, we assessed factors associated with all causes of diarrhea as a single outcome, which could have obscured risk factors unique to specific etiologies of diarrhea. Subgroup analysis of factors associated with cholera and non-cholera diarrhea was limited by sample size and insufficient power. Further research with a larger sample size would be beneficial to further our understanding of the factors associated with individual etiologies of diarrhea in this setting.

Another limitation is that self-reported data and missing data could each introduce bias to the study. Many of the variables were self-reported by the survey respondent, which could result in recall bias. However, prior analyses of these data did not find strong evidence of recall bias in self-reported vaccination data [17, 41]. Water treatment practices were among the self-reported data, and the absence of microbiological indicators of water quality limits interpretation of these data, which may also be susceptible to social desirability bias [75]. Missing responses also limit interpretation; if respondents did not know the child’s nutritional history, those responses were left blank, leading to missing data. In order for complete case analysis and missing indicator analysis to be valid, these missing data would need to be missing completely at random. The missing indicator variables for vitamin A and breastfeeding were not associated with the outcome of diarrhea, suggesting that these missing data were at least independent of the outcome. The inverse association between diarrhea and both exclusive breastfeeding and vitamin A remained when missing data were analyzed as an absence of exposure, suggesting a limited effect of bias caused by missing data for these variables. Missing data regarding zinc supplementation did appear to be associated with an elevated risk of diarrhea, although confidence intervals were wide. If having a missing value for this variable was associated with a child’s true zinc supplementation status, this could result in an inverse association between zinc and diarrhea as an artifact of selection bias. Another consideration is that if any controls had diarrhea without seeking treatment, this could bias the results toward the null, although major public health and community initiatives were underway at the time of the study to ensure access to treatment for diarrhea in both community and facility-based settings because of the cholera epidemic, so we believe this was unlikely to be a common occurrence. Finally, our findings represent associations between independent variables and diarrhea risk but do not necessarily indicate causal relationships.

In conclusion, we found that longer duration of exclusive breastfeeding and supplementation with vitamin A and zinc were inversely associated with diarrhea among children aged one to five years old in the context of a cholera epidemic in Haiti. Given that less than half of children under age six months are exclusively breastfed in Haiti, these findings highlight the importance of improving supports for breastfeeding mothers and addressing barriers to breastfeeding. The benefits associated with vitamin A and zinc supplementation, in the context of the high prevalence of micronutrient deficiencies among children in Haiti, emphasize the role of nutritional inadequacy in compounding diarrheal risk. Our results underscore the importance of integrated public health campaigns, which include identifying and implementing ways to support breastfeeding and address nutritional deficiencies, to reduce cholera and other diarrheal illness in children as part of comprehensive, multisectoral approaches to disease control.

## Supporting information

S1 Table Multivariable analysis of select variables with missing data using missing indicator method. (DOCX)

S2 Table Multivariable analysis of select variables with missing data when missing values are considered negative for exposure of interest. (DOCX)

S3 Table Interview questions on knowledge of cholera, sanitation and hygiene. (DOCX)

S4 Table Interview questions on micronutrient supplementation and breastfeeding. (DOCX)
