# Supplementary material for: Factors associated with diarrheal disease among children aged 1–5 years in a cholera epidemic in rural Haiti
Source: PLoS Negl Trop Dis. 2021 Oct 22;15(10):e0009726. doi: 10.1371/journal.pntd.0009726 (PMC8535179; doi:10.1371/journal.pntd.0009726)
Supplement: S1 Table — (DOCX) [file pntd.0009726.s001.docx]

Supporting information

| S1 Table. Multivariable analysis of select variables with missing data using missing indicator method | | | | | | | | |
| --- | --- | --- | --- | --- | --- | --- | --- | --- |
|  | Cases  (N=47) n (%) or  median (IQR) | | Controls  (N=166) n (%) or  median (IQR) | | Unadjusted  RR (95% CI) and P Value | | Adjusted RR (95% CI) and P Value ¶ | |
| Received Vitamin A within last 6 months | 4 | (9%) | 28 | (17%) | 0.32 (0.09 - 1.18) | 0.09 | 0.42 (0.11 - 1.63) | 0.21 |
| Missing vitamin A response | 19 | (40%) | 66 | (40%) | 0.71 (0.30 - 1.68) | 0.43 | 0.74 (0.29 - 1.90) | 0.53 |
| Received Zinc within last 6 months | 0 | (0%) | 15 | (9%) | † | † | † | † |
| Missing zinc response | 28 | (60%) | 76 | (46%) | 1.73 (0.63 - 4.72) | 0.29 | 2.19 (0.75 - 6.34) | 0.15 |
| Duration of exclusive breastfeeding In months (Median and IQR)*^a^* | 3 | (1-6) | 3 | (1-6) | 0.78 (0.58 - 1.06) | 0.11 | 0.79 (0.58 - 1.10) | 0.16 |
| Exclusive breastfeeding for ≤ 1 month | 11 | (41%) | 26 | (29%) | 3.56 (1.02 - 12.43) | 0.05 | 6.5 (1.42 - 30.06) | 0.02 |
| Missing breastfeeding responses | 1 | (4%) | 2 | (2%) | 0.73 (0.06 - 8.7) | 0.8 | 0.84 (0.06 - 11.18) | 0.9 |
|  |  |  |  |  |  |  |  |  |
| ¶ Adjusted for age (years), respondent relationship with participant, home has earthen floor, self-reported vaccination status. Adjusted analysis includes all 47 cases and 166 controls unless otherwise indicated. | | | | | | | | |
| *a* Questions related to breastfeeding apply only to children aged three and under. Adjusted analysis includes all 27 cases and 91 controls. | | | | | | | | |
