# Supplementary material for: Factors associated with diarrheal disease among children aged 1–5 years in a cholera epidemic in rural Haiti
Source: PLoS Negl Trop Dis. 2021 Oct 22;15(10):e0009726. doi: 10.1371/journal.pntd.0009726 (PMC8535179; doi:10.1371/journal.pntd.0009726)
Supplement: S2 Table — (DOCX) [file pntd.0009726.s002.docx]

Supporting information

| S2 Table. Multivariable analysis of select variables with missing data when missing values are considered negative for exposure of interest | | | | | | | | |
| --- | --- | --- | --- | --- | --- | --- | --- | --- |
|  | Cases  (N=47) n (%) or  median (IQR) | | Controls  (N=166) n (%) or  median (IQR) | | Unadjusted  RR (95% CI) and P Value | | Adjusted RR (95% CI) and P Value ¶ | |
| Received Vitamin A within last 6 months | 4 | (9%) | 28 | (17%) | 0.36 (0.10 - 1.29) | 0.12 | 0.49 (0.14 - 1.75) | 0.27 |
| Received Zinc within last 6 months | 0 | (0%) | 15 | (9%) | † | † | † | † |
| Duration of exclusive breastfeeding in months (Median and IQR)*^a^* | 3 | (1-6) | 3 | (1-6) | 0.79 (0.59 - 1.06) | 0.11 | 0.80 (0.58 - 1.09) | 0.16 |
| Exclusive breastfeeding for ≤ 1 month*^a^* | 11 | (41%) | 26 | (29%) | 3.14 (0.98 - 10.03) | 0.05 | 4.95 (1.27 - 19.29) | 0.02 |
|  | | | | | | | | |
| ¶ Adjusted for age (years), respondent relationship with participant, home has earthen floor, self-reported vaccination status. Adjusted analysis includes all 47 cases and 166 controls unless otherwise indicated. | | | | | | | | |
| *a* Questions related to breastfeeding apply only to children aged three and under. Adjusted analysis includes all 27 cases and 91 controls. | | | | | | | | |
