# Supplementary material for: Factors associated with diarrheal disease among children aged 1–5 years in a cholera epidemic in rural Haiti
Source: PLoS Negl Trop Dis. 2021 Oct 22;15(10):e0009726. doi: 10.1371/journal.pntd.0009726 (PMC8535179; doi:10.1371/journal.pntd.0009726)
Supplement: S3 Table — (DOCX) [file pntd.0009726.s003.docx]

­­­Supporting information

| S3 Table. Interview questions on knowledge of cholera, sanitation and hygiene | | | |
| --- | --- | --- | --- |
| **Question - English** | **Question - Kreyol** | **Coded Responses: Kreyol** | **Coded Responses: English** |
| How can a person get cholera? (Multiple answers can be selected) | Kòman moun ka trape maladi kolera? (yo ka chwazi plizye repons) | Bwè dlo ki pa trete = drinkuntreated; Manje manje ki pa byen kwit epi ki gen jèm nan = uncookedfood; Manje bèt ki sot nan lanmè ki pa kwit epi ki gen jèm nan = seafood; Manje fwi ki pa lave ak dlo trete = untr_wat_fruit; Bwè bwason nan lari ki pa fèt ak dlo trete = untr_wat_drink; Men sal = dirtyhands; Lot = other; Pa konnen = dontknow | drink untreated water; eat food that is not cooked well and has the germ; eat seafood that is not cooked well and that has the germ in it; eat fruit that is not washed with treated water; drink drinks that are no made with treated water; dirty hands, other |
| If yes, how can a person avoid getting cholera? (Multiple answers can be selected) | Kisa nou dwe fè pou nou ka evite gen maladi kolera? (yo ka chwazi plizye repons) | Lave men nou souvan ak dlo trete ak savon sitou lè n sot nan twalèt = washhands; Trete dlo n ap sèvi = treatwater; Pa kite fatra bò kote nap viv = nogarbage; Pa fè bezwen bò sous dlo = notoiletnearwat; Pa manje fwi ak legim kri ki pa lave ak dlo trete; nounwashedveg; Manje manje cho = hotfood; Vaksen = vaccine; Lot = other; Pa konnen = dontknow | washing hands often with treated water and soap, especially after going to the toilet; treating the water that we use; not leaving garbarge near where live; not going to the toilet near water source; no eating fruit/vegetables that are not washed with treated water; eating hot food; other |
| What are the ways to treat water that you drink? (Multiple answers can be selected) | Eske nou konnen kijan nou ka trete dlo nap bwè a? (yo ka bay plizye repons) Hint: Pa bay repons lan; kite moun nan reponn | Bouyi li = boil; Mete klowòks = chlorine; Mete solisyon ki pou pirifye li = otherpurifier; Mete grenn pou pirifye li = tablets; Lòt bagay =other; Pa konnen = dontknow | 1= boil it; 2= put chlorine; 3= put other solution to purify it; 3= put tablets to purify it; 4 = other |
| When should you wash your hands? (Multiple answers can be selected) | Eske ou ka di mwen kilè yon moun ta sipoze lave men li? (yo ka chwazi plizye repons) Hint: Pa bay repons lan; kite moun nan reponn | Anvan manje = before eating; Anvan prepare manje a = before preparing food; Anvan e aprè ou finn kenbe yon ti bebe = before and after handling babies; Aprè ou finn manyen bagay tankou lajan, telefòn nan, elatriye = after touching things like money, the telephone, etc; Aprè ou gen kontak ak yon moun ki gen vomisman oswa dyare = after handling a person with vomiting or diarrhea; Aprè ou finn twalèt = after going to the toilet; Lòt = other | before eating; before preparing food; before and after handling babies; after touching things like money the telephone etc; after handling a person with vomiting or diarrhea; after going to the toilet; other |
