# Supplementary material for: Factors associated with diarrheal disease among children aged 1–5 years in a cholera epidemic in rural Haiti
Source: PLoS Negl Trop Dis. 2021 Oct 22;15(10):e0009726. doi: 10.1371/journal.pntd.0009726 (PMC8535179; doi:10.1371/journal.pntd.0009726)
Supplement: S4 Table — (DOCX) [file pntd.0009726.s004.docx]

Supporting information

| S4 Table. Interview questions on micronutrient supplementation and breastfeeding | |  |  |
| --- | --- | --- | --- |
| **Children age 5 and under** |  |  |  |
| Did the child receive Vitamin A in the last 6 months? | Eske timoun nan te resevwa Vitamin A nan 6 mwa ki sot pase yo? | Wi=1; Non=0; Pa Konnen=dontknow | yes=1; no=0; .=unknown |
| Did the child receive zinc in the last 6 months? | Eske timoun nan te resevwa Zinc nan 6 mwa ki sot pase yo? | Wi=1; Non=0; Pa Konnen=dontknow | yes=1; no=0; .=unknown |
| **Children age 3 and under** |  |  |  |
| Is the child currently breastfed? | Eske pitit la tete toujou? | Wi=1; Non=0 | yes=1; no=0; .=unknown |
| What age (in months) was this child when s/he stopped breastfeeding? | Ki laj (an mwa) pitit la te genyen lè li te fè sevraj? | [number] | [number] |
| Until what age (in months) was the child exclusively breastfed? | Jiska ki laj (an mwa) timoun nan tete san li pa pran okenn lòt bagay ni pou bwè ni pou manje (sof lèt manman li)? | [number] | [number] |
